# A Low Computational Cost Algorithm for REM Sleep Detection Using Single Channel EEG

**DOI:** 10.1007/s10439-014-1085-6

**Published:** 2014-08-12

**Authors:** Syed Anas Imtiaz, Esther Rodriguez-Villegas

**Affiliations:** Department of Electrical and Electronic Engineering, Imperial College London, London, UK

**Keywords:** REM, Sleep staging, EEG, Electroencephalography, Rapid eye movement, Spectral edge frequency (SEF)

## Abstract

The push towards low-power and wearable sleep systems requires using minimum number of recording channels to enhance battery life, keep processing load small and be more comfortable for the user. Since most sleep stages can be identified using EEG traces, enormous power savings could be achieved by using a single channel of EEG. However, detection of REM sleep from one channel EEG is challenging due to its electroencephalographic similarities with N1 and Wake stages. In this paper we investigate a novel feature in sleep EEG that demonstrates high discriminatory ability for detecting REM phases. We then use this feature, that is based on spectral edge frequency (*SEF*) in the 8–16 Hz frequency band, together with the absolute power and the relative power of the signal, to develop a simple REM detection algorithm. We evaluate the performance of this proposed algorithm with overnight single channel EEG recordings of 5 training and 15 independent test subjects. Our algorithm achieved sensitivity of 83%, specificity of 89% and selectivity of 61% on a test database consisting of 2221 REM epochs. It also achieved sensitivity and selectivity of 81 and 75% on PhysioNet Sleep-EDF database consisting of 8 subjects. These results demonstrate that *SEF* can be a useful feature for automatic detection of REM stages of sleep from a single channel EEG.

**Electronic supplementary material:**

The online version of this article (doi:10.1007/s10439-014-1085-6) contains supplementary material, which is available to authorized users.

## Introduction

Human sleep is broadly classified in two groups: rapid eye movement (REM) and non-rapid eye movement (NREM). According to the American Academy of Sleep Medicine (AASM) sleep scoring manual, NREM stage is further divided in to N1, N2 and N3 stages with the progression of sleep.^20^ The standard method of sleep analysis is known as polysomnography (PSG), where several physiological signals are acquired, visually analyzed by sleep technicians and scored in to various stages. Manual analysis and scoring of sleep from PSG traces (acquired in clinic or at home) is a tedious task that can take 2–4 h for scoring an entire night sleep data.^35^ It is also prone to subjectivity between scorers with an inter-rater agreement of 82%.^10^ An automatic sleep staging method would help alleviate both inter-rater and intra-rater disagreements, reduce analysis time and cost of PSG tests.

The costs associated with PSG coupled with the necessity of clinical admission and long waiting lists^15^ limits its usage despite the high prevalence of sleep disorders.^38^ Home polysomnography (HPSG), classified as a type 2 portable monitoring device by AASM,^6^ offers full unattended PSG at patient’s home. It has recently been shown to be useful to rule in or out obstructive sleep apnea (OSA), results in better sleep quality of patients and reduced overall costs.^4^ HPSG requires at least seven channels including multiple EEG, EOG and EMG channels. The complexity imposed by the requirement of the patient precisely placing these multiple electrodes limits the adoption of HPSG despite its benefits.

Thus, HPSG systems would greatly benefit from reduction in the number of channels, simplification of user experience and incorporation of automated sleep scoring methods without affecting clinical outcomes. Traditionally, three EEG channels are required in PSG systems together with EOG and EMG channels. Ruehland* et al.* 36 reported no significant differences in sleep scoring reliability when using a single EEG channel, so this number can potentially be reduced to one. However, the EOG and EMG channels are still required since identifying REM stage epochs involves observing the chin muscle and eye activity.^20^

REM sleep accounts for about 5–20% of an adult’s entire night’s sleep^19^ and its detection, both onset and duration, are very important for the diagnosis of certain sleep disorders including narcolepsy and REM behavior disorder (RBD). Observing the muscle activity during REM stage is often used for the diagnosis of RBD, which is also an early marker for neurological disorders including Parkinson’s disease.^22^ The duration of REM sleep in the first cycle has been shown to correlate negatively with mood improvement on wake-up in patients with major depression.^21^ It has also been shown that the number of REM sleep periods is higher, with a shorter average duration, in trauma-exposed people who go on to develop posttraumatic stress disorder.^29^ The latency to the onset of first REM cycle and the pattern of occurrence of subsequent cycles throughout the night is commonly used in the diagnosis of narcolepsy. Vogel* et al.* 43 reported that REM sleep deprivation can be used therapeutically for the improvement of depression symptoms. Using a wearable REM sleep detection system, this can be achieved by raising an alarm to awaken the person whenever they enter the REM phase. REM deprivation can also result in increased alertness during daytime.^30^

Apart from REM, all the stages of sleep can be identified from EEG channels only. This is because REM sleep has many electroencephalographic similarities with Wake and N1 stages.^3^,^9^,^19^ According to both R&K^34^ and AASM^20^ sleep scoring manuals, the presence of low amplitude, mixed frequency EEG is characteristic of both N1 and REM stages making its visual identification using EEG challenging. However, with most of the sleep stages identifiable with EEG it makes sense to attempt to score REM phases using the same signal to obviate the need of using extra electrodes.

A reduction in the number of channels also leads to smaller processing overhead for portable and wearable systems with weight, size and power consumption limits, as there is less data to acquire and process. In such systems, algorithms for scoring sleep are also constrained by the processor since complex processing methods directly result in higher power consumption and a reduced battery life of the system. The two key stages in all sleep staging methods are feature extraction and classification. The number and types of features extracted and the choice of classifier used depends on the target application of an algorithm. For example, it may be acceptable to use 20 features with a multistage neural network in an analysis software running on a computer but the limitations of a wearable battery-powered system prohibits the usage of complex features and classifiers consequently leading to a reduction in performance. Therefore a trade-off between acceptable levels of performance and algorithm complexity must be made to meet system specifications.

This paper has two main objectives. The first is to find features and trends in sleep EEG that can distinguish REM phase from all other stages of sleep, particularly N1 and Wake. The second objective is to use these EEG features for developing a simple algorithm capable of detecting REM stage epochs. Both these objectives ultimately aid the development of an algorithm that could be used as part of a truly wearable full sleep staging system. The remainder of this section presents a review of various automatic sleep staging methods and their REM detection performance. “Material and Methods” section describes the sleep data used for the development of algorithm in this work, introduces the individual features and discusses their discriminatory ability for REM detection. The features are then combined to develop a complete REM detection algorithm which is presented in the same section. The performance results are presented in “Results” section. “Discussion” section discusses the effectiveness of the features used, overall results and the advantages of the proposed features and algorithm.

Several research groups have been working on automatic sleep staging using signals from PSG and EEG based systems. In this section a review of these methods is presented to show the different features and classifiers being used and their detection performance. The performances reported below are limited to the REM detection part of systems and their corresponding accuracy.

Agarwal and Gotman^1^ used a computer-assisted approach requiring an expert reviewer input to score sleep stages. They used two EEG and EOG channels with one channel of EMG signal to compute several features including spectral power ratio in different frequency bands, eye movements and dominant rhythm with k-means clustering for classification. Their method was tested with 12 subjects having 2519 REM epochs and showed sensitivity and specificity of 72.5 and 87.6% respectively in REM stage. Virkkala* et al.* 42 used facial electrodes for acquiring signals and tested their sleep staging method on 131 subjects having 24021 REM epochs. Their system used a decision tree classifier and resulted in REM stage sensitivity and selectivity of 61.6 and 79.4% respectively. Liang* et al.* 26 used a decision tree with power and energy features followed by contextual smoothing for sleep staging. Their method used single channel EEG and EMG with two channels of EOG signals and resulted in REM stage sensitivity and specificity values of 90.5 and 95.5%. They also presented a single-channel EEG-based method^27^ using multiscale entropy (MSE) and autoregressive (AR) modeling. They used a total of 21 MSE and AR features with LDA classifier and 11 contextual smoothing rules and reported REM sensitivity and selectivity of 97.6 and 95.6% on 10 test subjects. Held* et al.* 18 presented a neuro-fuzzy classifier based infant sleep staging method using four EEG, one EMG and one EOG channels and reported REM stage detection sensitivity of about 72% with 250 REM epochs in their test set. A study evaluating the performance of an automatic sleep staging software (ASEEGA) using single channel EEG reported REM sensitivity and selectivity values as 83 and 89.1% respectively^2^ for five state sleep classification. The algorithm worked by performing artefact rejection, extracting multiple spectral and temporal features, identifying sleep microstructure and performing rough REM detection using theta, beta and delta rhythms. This is followed by the use of a fuzzy classifier and contextual rule smoothing with a fixed set of rules. Although the software achieves a high detection performance (on artefact-free signals), this comes at the cost of computational complexity during the feature extraction and fuzzy classification stages.

Hanaoka* et al.* 17 proposed a sleep staging system that used EEG, EOG and EMG signals for feature extraction and decision tree learning for classification. For REM detection, it checked for ocular movement and low EMG activity. The algorithm was tested on eight hours of PSG recording from only one subject containing a total of 215 REM epochs and resulted in a sensitivity of 75.5%. Kempfner* et al.* 23 used eighteen statistical features with subject-specific feature scaling and k-Nearest Neighbor classifier to detect REM sleep in subjects without atonia. They used inputs from two EOG and three EEG channels and reported mean sensitivity and specificity of 94 and 96% respectively with 16 test subjects.

Artificial neural networks (ANN) are commonly used for sleep stage classification. Methods using these networks often require a large set of temporal, spectral and statistical features to be extracted from the input signal. hese features are then given as inputs to the network which maps them to discrete sleep stages. Due to this, ANNs are computationally expensive and require powerful processors that may be detrimental for battery-powered wearable devices with limited power budget. Ronzhina* et al.* 35 describe a method for sleep staging using single channel EEG with an ANN architecture comprising of 30 input units and 11 hidden layer units. The authors used relative power values in 30 spectral bands of 1 Hz each and reported the best REM stage accuracy of 82.3% on data from 8 subjects. A hybrid neural network based method, proposed by Park* et al.*,^31^ used 58 input features extracted from EEG, EOG and EMG signals. It was tested with only 218 REM epochs and resulted in 212 correct detections. Another method using neural networks for sleep staging using EEG and EMG signals is reported by Tian* et al.* 39 with 84.8% sensitivity when tested on 1278 REM epochs. Charbonnier* et al.* 5 also used ANN with 33 spectral, entropy and statistical features. They reported REM sensitivity to be 63% using EEG signals only. They also showed that adding EMG signals increased REM stage sensitivity up to 83%. Ebrahimi* et al.* 11 used wavelet packet coefficients extracted from a single EEG channel as input features for a neural network. For a combined detection performance of N1 and REM stages they reported sensitivity and specificity values of 85.7 and 93.8%. They did not state how many REM or N1 were detected individually and tested their method with data from 7 subjects having 1252 REM epochs in total. Gunes* et al.,* 16 in their single channel EEG based sleep staging method used k-means clustering and k-Nearest Neighbor classifier. They reported REM stage sensitivity of 81% when tested with 600 epochs. A study validating the performance of a commercial wireless sleep monitoring system^37^ used 26 subjects with 3036 REM epochs and reported sensitivity and selectivity as 86 and 74% respectively. The sleep system in this study used three electrodes in a headband for acquiring signals. (This device, ZEO sleep manager, is not available any more because the company went out of business in early 2013).

Estrada* et al.* 13 concluded that EMG and EOG are both important in sleep staging, particularly in REM stage. Similarly, Charbonnier* et al.* 5 reported a jump in REM detection accuracy from 63 to 83% when EMG signal was added to their analysis. It is evident from the sleep staging literature that algorithms using inputs from EEG, EOG and EMG channels are able to achieve a better REM detection performance while using just one EEG channel makes the task more challenging.

## Material and Methods

Twenty whole night PSG recordings of healthy subjects were available in the DREAMS Subjects Database from University of MONS—TCTS Laboratory and Université Libre de Bruxelles—CHU de Charleroi Sleep Laboratory in EDF format.^41^ The subjects included 16 women and 4 men, their age ranging from 20 to 65 years (mean age 33.45 years). Data was originally sampled at a frequency of 200 Hz and included at least two EOG, three EEG (Fp1-A2, Cz-A1 and O1-A2) and one submental EMG channels. The epochs were scored using AASM^20^ criteria with standard epoch size of 30 s.

Before being used for any analysis data from each of the EEG channel was first resampled to a sampling frequency of 256 Hz using Matlab *resample* function. The signal was then filtered with a first order 0.16 Hz high pass filter to remove dc offset and a second order 50 Hz Butterworth low pass filter to bandlimit it. From the pool of twenty subjects, the first five (subjects 1–5) were arbitrarily selected for data analysis, feature selection and training of the proposed algorithm. Subjects 6–20 were later used to test the performance of the algorithm without any parameter adjustment. The total number of epochs in Wake, REM and NREM stages for the training and test set are shown in Table 1.

**Table 1:** The number of Wake, REM and NREM epochs in training and test set

	No. of subjects	Number of epochs		
		Wake	NREM	REM
Training	5	679	3573	798
Test	15	2880	10091	2221

Corsi-Cabrera* et al.* 9 reported similar N1 and REM spectral powers between 13 and 17 Hz, higher N1 power in the 10–13 Hz band and lower N1 power between 1 and 9 Hz. Uchida* et al.* 40 also showed spectral power in REM to be lowest in the 12–16 Hz band when compared to NREM stages (except N1). Since the 10–13 Hz band appears to be able to discriminate REM and N1 while 12–16 Hz helps distinguishing REM from other stages, we performed our analysis in these as well as other frequency bands. This is done to determine the best frequency range where the discriminatory ability of different features are most prominent. To this end, we selected a frequency range of 8–16 Hz for our analysis (based on results shown later) and also compared the performance of same features in this band against the traditional 0.5–50 Hz range.

**Figure 1:**
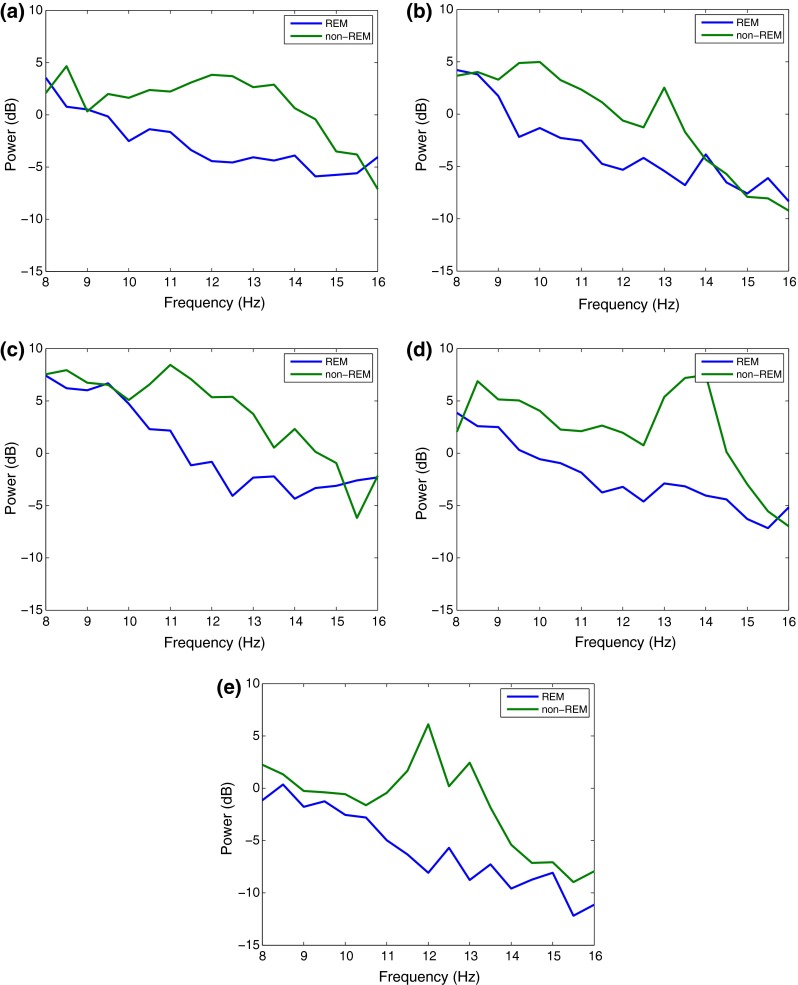
Frequency spectrum of REM and non-REM epochs in 8–16 Hz range for different training subjects 1–5 on plots (a–e)

The EEG data was split into 2-s long non-overlapping blocks (subepochs) and subsequently transformed to the frequency domain with a 512-point fast Fourier transform (FFT), hence obtaining a resolution of 0.5 Hz. The magnitude and frequency coefficients were then used to compute the following features for REM detection in both the 8–16 Hz and traditional frequency bands. The frequency spectrum for REM and non-REM epochs in the 8–16 Hz range is shown in Fig. 1. The differences in power at different frequency bands will be analyzed in the following sections.

### Spectral Edge Frequency (SEF)

**Figure 2:**
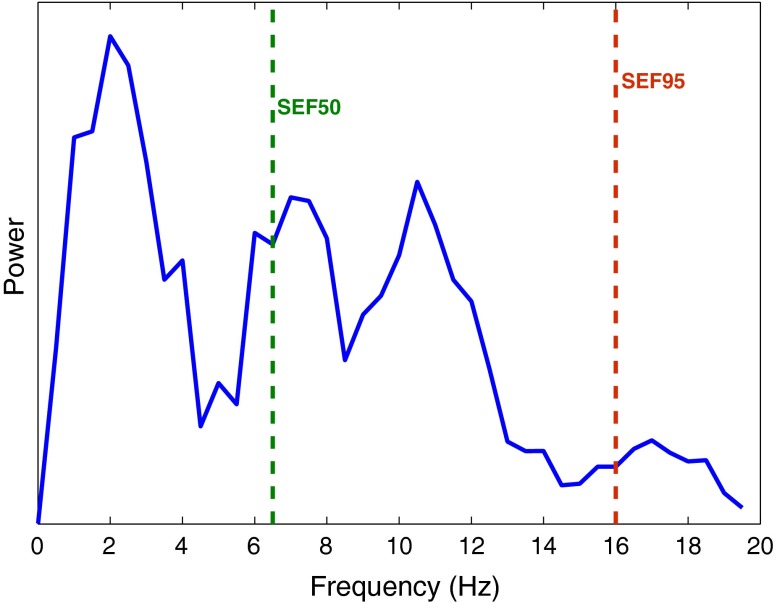
An illustration of spectral edge frequency (*SEF*) at 50 and 95% of the signal power in the 0–20 Hz frequency range

Spectral edge frequency (*SEF*) is the frequency below which a certain fraction of the signal power is contained. It is generally written as *SEFxx* where *xx* is the fraction of signal power for which the edge frequency is calculated. An illustration of spectral edge frequency at 50 and 95% of the signal power is shown in Fig. 2.

Three different quantifications of *SEF* are relevant for this work:

#### SEF50

*SEF* at 50% (*SEF50*) is the lowest frequency below which half of the signal power is present. This is equivalent to the median frequency of a signal. It is computed from the FFT coefficients using Eq. (1), where* n* is the total number of FFT coefficients and* x* is the index to solve the equation for. The required frequency is then the* x*th frequency from the array of FFT frequency components.

1a $$\begin{aligned} \sum \limits _{i=1}^x |{\text{mag}}_{i}|^2 = 0.50 \times \sum \limits _{i=1}^n |{\text{mag}}_{i}|^2\end{aligned},$$

1b $$\begin{aligned} SEF50 = {\text{freq}}(x) \end{aligned}.$$

**Figure 3:**
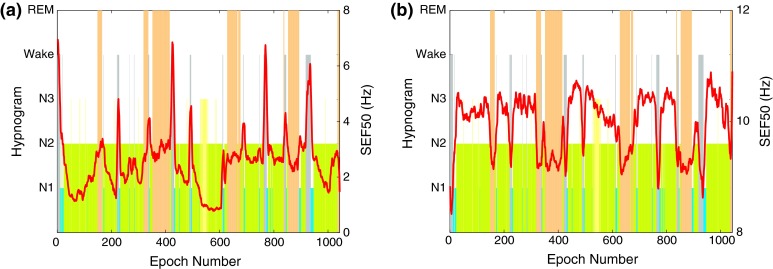
Hypnogram and *SEF50* in the (a) 0.5–50 Hz and (b) 8–16 Hz band of the EEG signal for one training subject

Figure 3 shows the hypnogram together with *SEF50* in the 0.5–50 and 8–16 Hz frequency bands. During the REM stages, the *SEF50* values are observed to be amongst the lowest when calculated in the 8–16 Hz range in Fig. 3b. However, this is not the case in Fig. 3a when the entire frequency range is used and the *SEF50* values during REM stages overlap with with those during N2 stages.

#### SEF95

*SEF* at 95% (*SEF95*) is the lowest frequency below which 95% of the signal power is present. It is computed from the FFT coefficients using Eq. (2), similar to the *SEF50* calculation.

2a $$\begin{aligned} \sum \limits _{i=1}^x |{\text{mag}}_{i}|^2 = 0.95 \times \sum \limits _{i=1}^n |{\text{mag}}_{i}|^2 \end{aligned},$$

2b $$SEF95 = {\text{freq}}(x).$$

**Figure 4:**
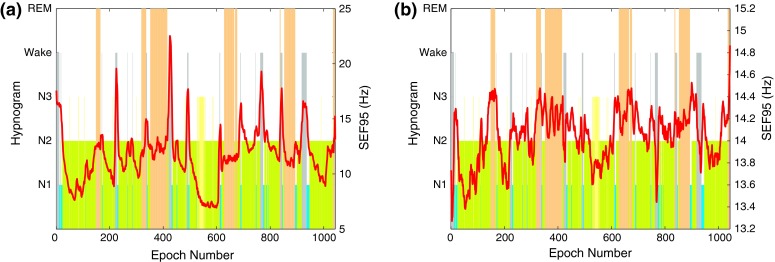
Hypnogram and *SEF95* in the (a) 0.5–50 Hz and (b) 8–16 Hz band of the EEG signal for one training subject

Figure 4 shows how *SEF95* varies in different sleep stages for one subject in the two frequency ranges. The *SEF95* values in the 0.5–50 Hz analysis range during REM stages are neither highest nor lowest and stay close to the 12 Hz mark. In the 8–16 Hz range, however, *SEF95* values are usually highest during the REM stages.

#### SEFd

The difference between *SEF95* and *SEF50* is used as a novel feature for REM stage detection in this work. This difference is hereon referred to as *SEFd*. For an epoch* e*, it is determined by first calculating the *SEFd* values of fifteen 2 s subepochs in the 30 s EEG epoch (*i.e.,* the difference between SEF95 and SEF50 of the subepochs). The mean of these differences is taken to be the *SEFd* of the epoch being processed as shown in (3) where* se* is the subepoch and* n* is its index. A 9-point moving average filter is then applied to the final *SEFd* value.

3 $$SEFd(e) = \frac{1}{15} \times \sum \limits _{n=1}^{15} (SEF95[se_{n}]-SEF50[se_{n}]).$$

**Figure 5:**
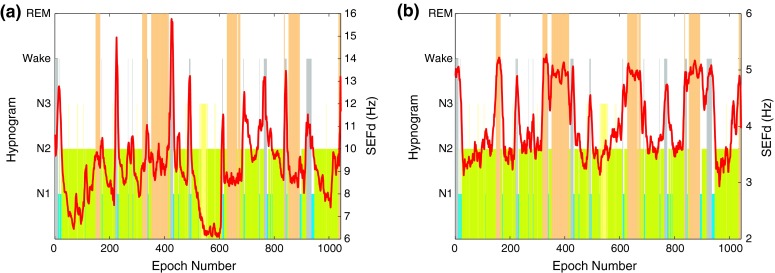
Hypnogram and *SEFd* in the (a) 0.5–50 Hz and (b) 8–16 Hz band of the EEG signal for one training subject

In Fig. 5 the *SEFd* values during different sleep stages are shown in both traditional and bandlimited frequency ranges. The figure shows clear peaks during REM stages when the analysis is restricted to the 8–16 Hz range. However no such characteristic pattern is observed when the entire frequency band is analyzed.

**Figure 6:**
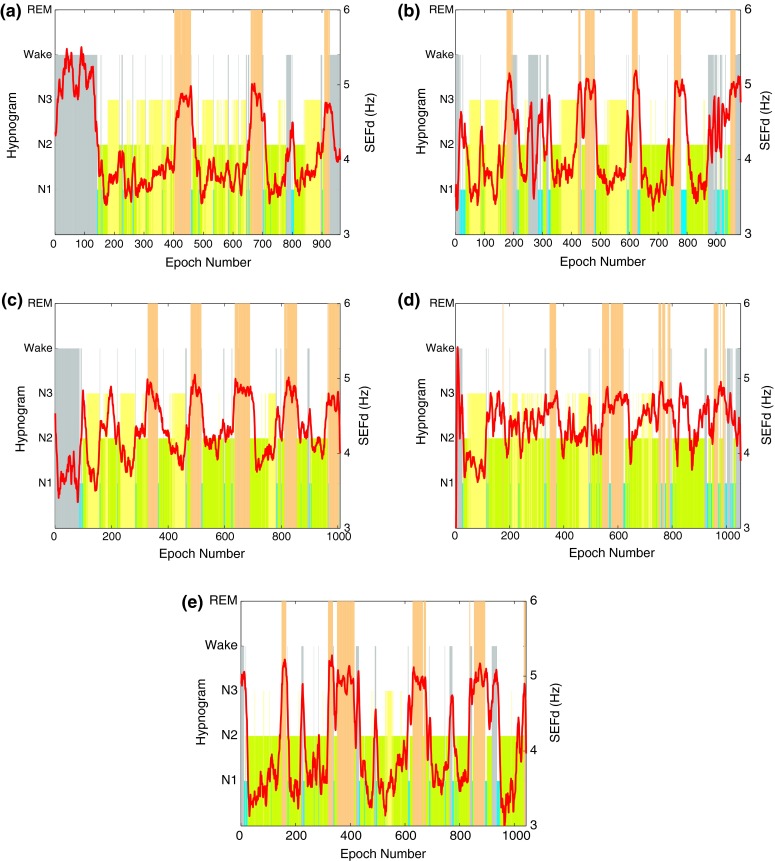
Hypnogram and *SEFd* in the 8–16 Hz band of the EEG signal for training subjects 1–5 on plots (a–e) respectively. The plots show clear peaks during all the REM phases for every case

Figure 6 shows the *SEFd* overlaid on the hypnogram of each training subject in the 8–16 Hz range and illustrates that the values of *SEFd* are consistently high during all REM phases for the entire sleep duration of all subjects. In general, all N2 and N3 phases appear to have lower *SEFd* values. N1 stages have a slightly higher value but still lower than REM stages in most cases. This pattern of high *SEFd* values during REM phase in the 8–16 Hz frequency band could be a useful feature to discriminate it from other sleep stages.

The reason for high *SEFd* values is a result of lower *SEF50* and higher *SEF95* values during REM stages. The two trends in *SEF* can be explained by the observations in Fig. 1 which shows how the power within the 8–16 Hz band changes during both REM and non-REM stages (including Wake). The power is similar in both REM and non-REM around 8 Hz. Following this, the power in REM is lower than non-REM from 9–15 Hz with the difference being highest around the 12 Hz mark. Uchida* et al.* 40 reported the absence of 12–16 Hz activity during REM stages which is causing the power to be lower than non-REM. Therefore the median frequency (*SEF50*) in 8–16 Hz range is expected to be lower during REM stages. The trend of higher *SEF95* values during REM suggests an increase in the higher frequency components of the 8–16 Hz band. In Fig. 1, apart from 1e, all cases demonstrate an increase in the power spectrum of REM around the 15 Hz mark. Further, the activity in the neighbouring beta frequency band is also highest during REM sleep.^40^ This causes the *SEF95* values to be higher during REM within the 8–16 Hz range. *SEFd* essentially represents both these changes in *SEF50* and *SEF95*, which is observed to be greatest when the frequency band is limited between 8 Hz and 16 Hz.

To quantify the discriminatory ability of *SEFd* as compared to both *SEF50* and *SEF95* features individually in the 8–16 Hz frequency range, all the three different features were used to classify REM epochs in both frequency ranges. A simple thresholding classifier was used and the receiver operating characteristic (ROC) curves was plotted in each case by sweeping the detection threshold. The area under the curve (AUC) for the three features in both frequency ranges, shown in Table 2, confirms that all features perform better when limited to the 8–16 Hz frequency range. Further, it also shows that *SEFd*, as a feature, is far superior to both *SEF50* and *SEF95* with a much higher AUC value. Therefore, *SEFd* in the 8–16 Hz band is used as the main feature for REM detection in this work.

**Table 2:** AUC values for the three features in different frequency ranges

Feature/ frequency range	0.5–50 (Hz)	8–16 (Hz)
*SEF50*	0.7023	0.7530
*SEF95*	0.7082	0.7390
*SEFd*	0.6930	0.9247

**Figure 7:**
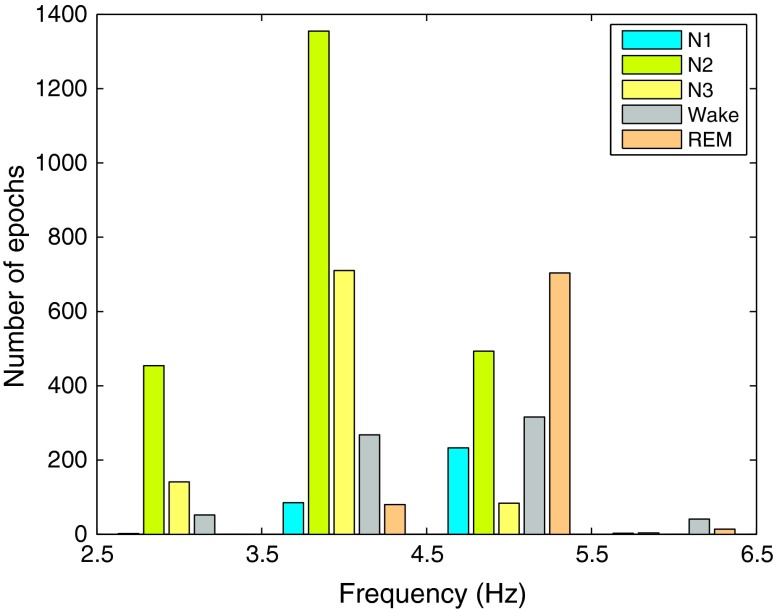
Frequency distribution of SEFd values at different sleep stages across all training subjects

The *SEFd* shows peaks during REM phases for all the subjects but occasional peaks are also observed during other phases of sleep in some cases. For example, subject 1, in Fig. 6a, shows high values of *SEFd* during Wake stage (similar to those during REM) while this is not the case for subject 3, in Fig. 6c. The frequency distribution plot for the training data in Fig. 7 also shows that while most of the REM epochs have *SEFd* values of more than 4.5 Hz, there are still some epochs from other stages overlapping in this frequency range. Due to this, two other features are also investigated to reduce potential false detections occurring in other sleep stages.

### Absolute Power (AP)

The absolute power (*AP*) of a signal in a fixed frequency range, $f_{1}-f_{2}$ Hz, using its Fourier coefficients is calculated using Eq. (4), where $f_{1}$ and $f_{2}$ are 8  and 16 Hz respectively and $n(f_{1})$ and $n(f_{2})$ are the indices at these frequencies.

4 $$AP = 20 \times {\text{log}}\left(\sum \limits _{i=n(f_{1})}^{n(f_{2})} |{\text{mag}}_{i}|\right).$$

**Figure 8:**
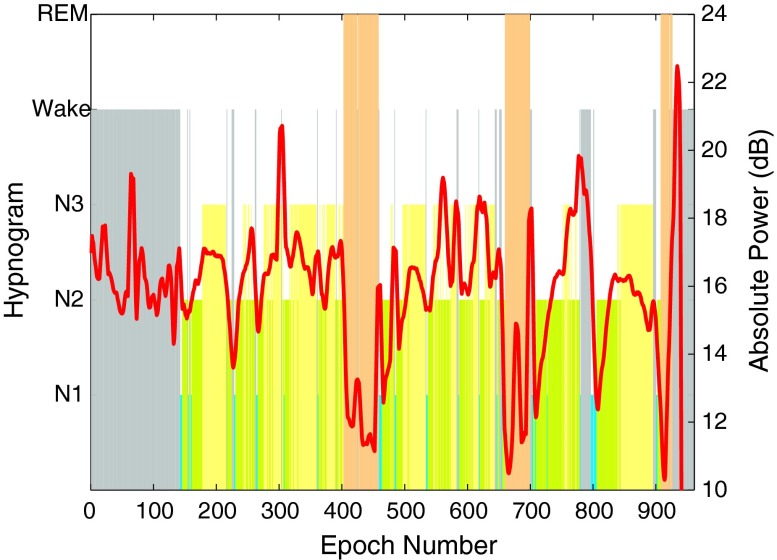
Hypnogram and *AP* in the 8–16 Hz band of the EEG signal for training subject 1. *AP* values can be seen to be lowest during each REM phase

*AP* was calculated using Eq. (4) for each 2 s subepochs and averaged over the standard 30 s epoch. Figure 8 shows the absolute power with hypnogram for subject 1. REM stage was observed to have the lowest *AP* in 8–16 Hz range. Further, *AP* values during Wake and N1 stages were higher than REM. These results are in line with the observations in Refs. 9 and 40. *AP* hence, could be used as an extra differentiating feature for REM, Wake and N1 stages. Similar trends were also observed for the other training subjects.

### Relative Power (RP)

The relative power (*RP*) of a signal in a fixed frequency range, $f_{1}-f_{2}$ Hz (8–16 Hz) is calculated, as in Eq. (5), by taking the ratio of the absolute powers of the signal in the range of interest and the entire signal bandwidth.

5 $$\begin{aligned} RP = 20 \times {\text{log}}\left( \frac{\sum \limits _{i=n(f_{1})}^{n(f_{2})} |{\text{mag}}_{i}|}{\sum \limits _{i=1}^{n} |{\text{mag}}_{i}|}\right) \end{aligned}.$$

**Figure 9:**
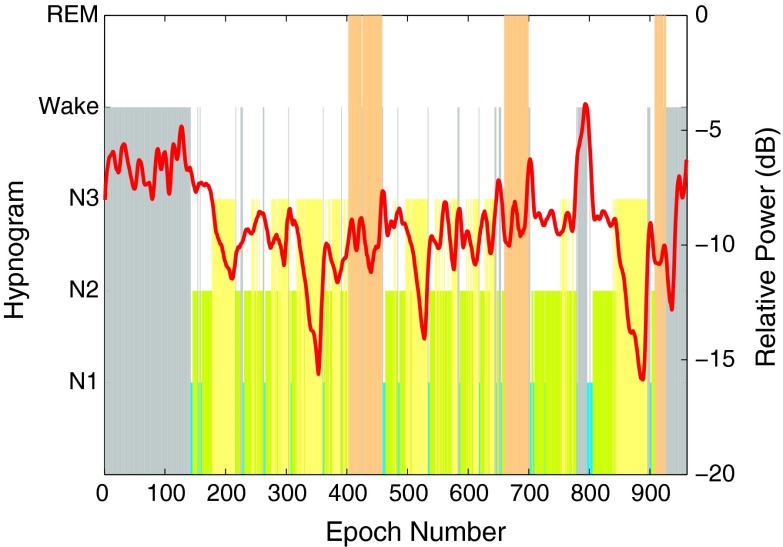
Hypnogram and *RP* in the 8–16 Hz band of the EEG signal for training subject 1. *RP* values can be seen to be stable around −20 dB mark

*RP* was also calculated first for 2 s subepochs and then averaged over 30 s epochs. Figure 9 shows the relative power in 8–16 Hz for subject 1 together with its hypnogram. During REM stage, *RP* does not exhibit any characteristic peak or trough unlike *SEFd* or *AP* plots. However, the values stay close to −8 dB range approximately for all subjects and are also different from those during N3 and Wake stages. This makes the feature useful for reducing potential false detections.

### REM Detection Algorithm

**Figure 10:**
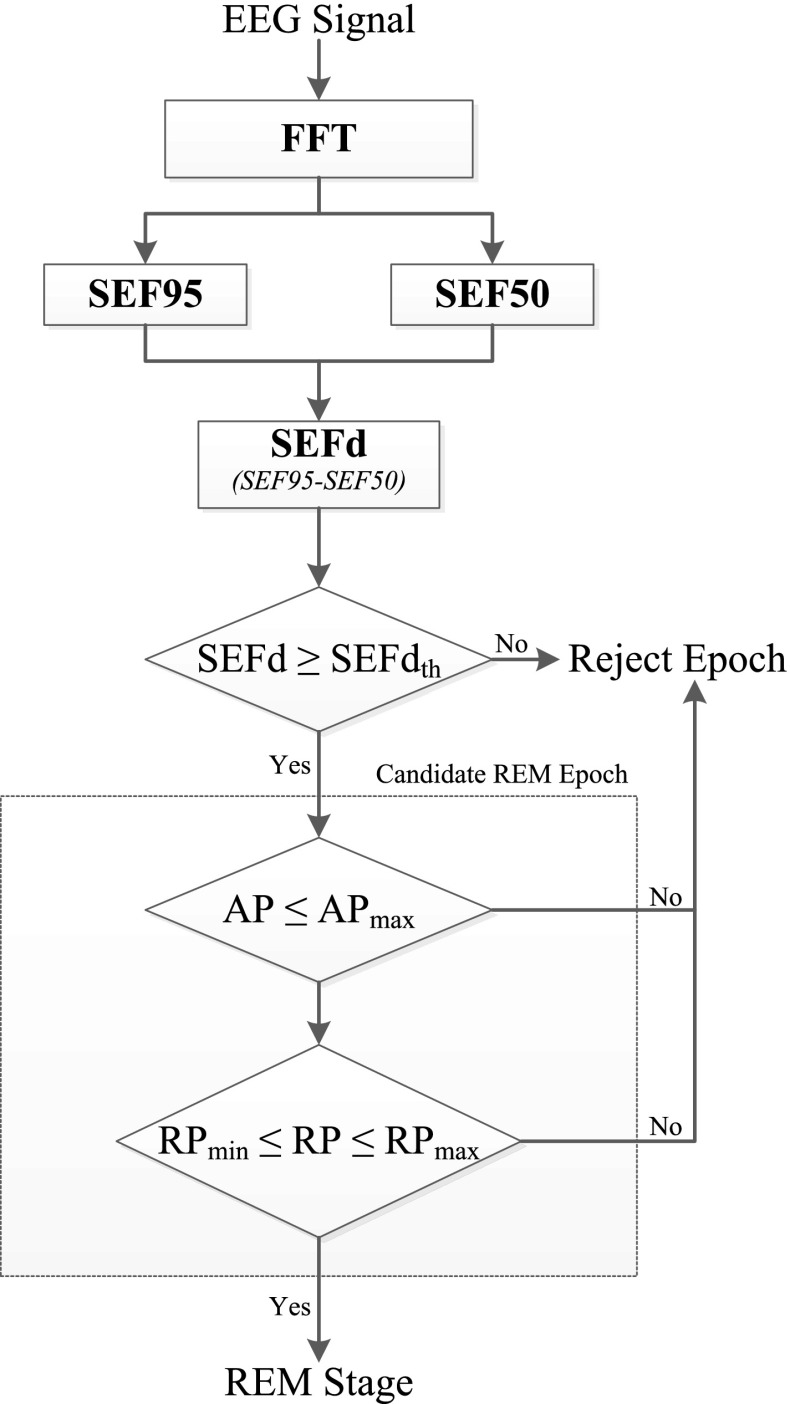
Block diagram of the REM detection algorithm

Figure 10 shows a complete flow chart of the proposed REM detection algorithm. A single channel EEG input is first transformed into the frequency domain using the FFT. In the first stage FFT coefficients are used to compute *SEF95* and *SEF50* within the 8–16 Hz band. The difference between these two spectral edge frequency measures, *SEFd*, is then taken for every epoch. If *SEFd* is found to be greater than a certain maximum threshold *SEFd*th, the epoch under analysis is marked as a candidate REM epoch (*cREM*), and further checks are applied at the next stage. Otherwise, the epoch is rejected as non-REM and not analyzed any further.

6 $$\begin{aligned} E(n)= {\left\{ \begin{array}{ll} cREM, &{} \text {if } SEFd(n) \ge SEFd_{th} \\ 0, &{} \text {otherwise} \end{array}\right. } \end{aligned}.$$

The second stage of the algorithm is used to reject false positives amongst the candidate REM epochs. If an epoch satisfies the condition in Eq. (6), its *AP* and *RP* values are evaluated in the 8–16 Hz range for further analysis.

7 $$\begin{aligned} AP \le AP_{\text{max}} \end{aligned},$$

8 $$\begin{aligned} RP_{\text{min}} \le RP \le RP_{\text{max}} \end{aligned}.$$

Only when both *AP* and *RP* values satisfy the conditions in Eqs. (7) and (8), a candidate REM epoch is considered a true detection. Otherwise it is rejected as non-REM.

The algorithm works in two stages where the first stage is highly sensitive and detects candidate REM epochs. The second stage is specific and helps in reducing the number of false detections. The choice of features used at each of the two stages was determined by their discriminatory ability in detecting REM epochs. *SEFd* was found to be the most sensitive feature and was therefore used at the first stage of the algorithm (to shortlist as many REM epochs as possible) followed by *AP* and *RP*. This two-stage process also helps in keeping the computational load low since *AP* and *RP* features are calculated only when there is a candidate REM epoch identified in the first stage.

## Results

### Metrics

The performance of the algorithm is evaluated by quantifying the following metrics.

1. Sensitivity, which represents the fraction of REM epochs that are correctly identified by the algorithm.
  9 $${\text{Sensitivity}} = \frac{TP}{TP+FN}$$
2. Specificity, which represents the fraction of non-REM epochs being correctly rejected.
  10 $${\text{Specificity}} = \frac{TN}{TN+FP}$$
3. Selectivity, which is the fraction of correct detections of REM with respect to the total number of automatic REM detections (also known as positive predictive value or PPV).
  11 $${\text{Selectivity}} = \frac{TP}{TP+FP}$$
4. Accuracy, which is the fraction of the total number of correct detections and rejections of REM epochs in the sleep recording.
  12 $${\text{Accuracy}} = \frac{TP+TN}{TP+FP+TN+FN}$$

In the equations above, true positives (*TP*) is the number of epochs correctly scored as REM, false positives (*FP*) is the number of epochs incorrectly scored as REM, true negatives (*TN*) is the number of epochs correctly rejected as non-REM, and false negatives (*FN*) is the number of epochs incorrectly rejected as non-REM.

### Training Results

**Figure 11:**
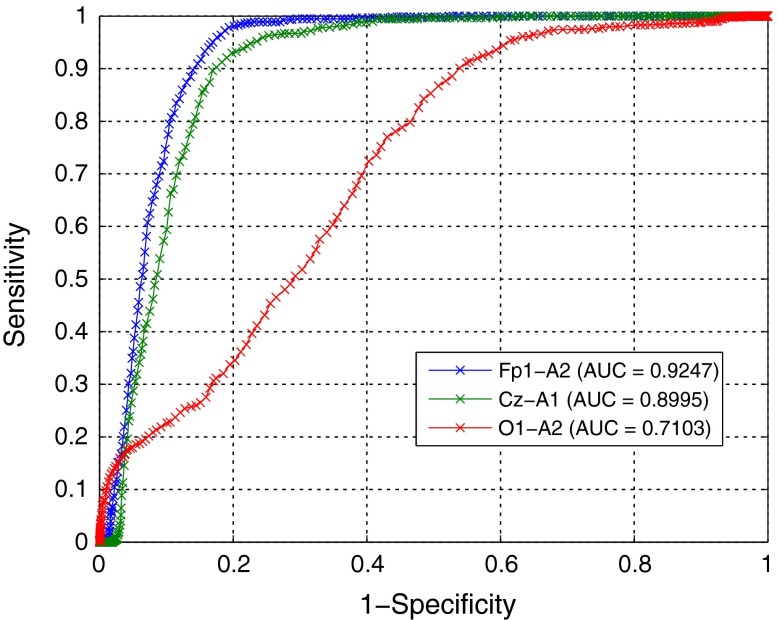
ROC Curves with AUC at first stage of algorithm for three EEG channels

Data from five subjects was used during the training stage of the algorithm. The detection thresholds (*SEF*th, *AP*_max_, *RP*_max_ and *RP*_min_) were tuned to achieve the best average performance. For this, a ROC curve was plotted of sensitivity against (1-specificity) with varying thresholds for the first stage initially. The ROC curves for three different EEG channels and the AUC for each are shown in Fig. 11.

Since the largest AUC is for channel Fp1-A2, it is selected as the one to use for further analysis. On the ROC curve, the optimal operating point for the first stage of the algorithm (*SEF*th) was established by giving equal weight to both sensitivity and specificity and determining the minimum distance of the curve from the (0,1) coordinate.^7^,^32^ This is the point on the curve closest to the (0,1) coordinate. Using this optimal threshold, the candidate REM epochs (with *SEFd* greater than this threshold) were analysed. For these epochs, a second ROC curve was plotted by sweeping the *RP* and *AP* thresholds. The optimal operating point for these features was also established by determining the shortest distance of the second curve from the (0,1) coordinate. The thresholds corresponding to the optimal points for both stages of the algorithm are shown in Table 3. It should be noted that a different operating point could be selected depending on whether higher sensitivity at the cost of more false positives is tolerable or if a lower false positive rate is desired at the cost of sensitivity.

**Table 3:** Best performing thresholds for *SEFd*, *AP* and *RP*

Parameter	Value
SEFth	4.54 Hz
AP_max_	15.5 dB
RP_max_	−6.08 dB
RP_min_	−13.03 dB

The algorithm individual as well as average subject performance using the fixed optimum thresholds is shown in Table 4. All the subjects showed sensitivity greater than 89% individually and around 94% on average. Only 46 out of the total 798 REM epochs were not detected by the algorithm while the number of false positives was recorded as 475 epochs from a total of 5050 epochs across all five subjects. Most of the Wake and NREM epochs were correctly rejected giving an average specificity of 89%. The overall accuracy of the system was found to be close to 90%.

**Table 4:** Performance of algorithm on training database

Subject	REM_tot_	REM_det_	TP	Sen (%)	Spe (%)	Se l(%)	Acc (%)
1	113	158	103	91.15	93.51	65.19	93.24
2	122	242	119	97.54	85.75	49.17	87.21
3	212	224	189	89.15	95.60	84.38	94.25
4	155	324	146	94.19	80.18	45.06	82.24
5	196	279	195	99.49	90.08	69.89	91.85
Total	798	1227	752				
Average				94.31	89.03	62.74	89.76

*REM*_tot_ number of REM epochs in the test,* REM*_det_ number of REM epochs detected by the algorithm.* TP* true positives,* Sen* sensitivity,* Spe* specificity,* Sel* selectivity,* Acc* accuracy

### Test Results

The algorithm was tested using the detection thresholds in Table 3 on complete night EEG recordings of 15 subjects. Results of the individual and average performance are shown in Table 5. The average sensitivity for these test subjects is reduced to 83%. Apart from subjects 12 and 14, all have a sensitivity of more than 70% and even in these cases where sensitivity is on the lower side, the accuracy is still greater than 92%. Subject 12, with the lowest sensitivity, has got a large Wake period in the middle of sleep and sporadic Wake epochs throughout the night. The exact cause of this Wake period is not known but it leads to the presence of movement artefacts, making the detection of REM difficult. The average specificity, selectivity and accuracy values of the test set are, however, similar to the training results.

**Table 5:** Performance of algorithm on test database

Subject	REM_tot_	REM_det_	TP	Sen (%)	Spe (%)	Sel (%)	Acc (%)
6	187	297	186	99.47	86.28	62.63	88.76
7	131	285	107	81.68	79.8	37.54	80.04
8	162	284	120	74.07	79.68	42.25	78.74
9	131	184	124	94.66	93.91	67.39	94
10	146	153	113	77.4	95.48	73.86	92.92
11	212	268	203	95.75	91.83	75.75	92.66
12	87	64	52	59.77	98.63	81.25	95.11
13	89	267	88	98.88	82.42	32.96	83.74
14	163	118	105	64.42	98.45	88.98	92.93
15	123	113	92	74.8	97.06	81.42	93.79
16	147	164	105	71.43	92.8	64.02	89.56
17	162	259	137	84.57	85.16	52.9	85.06
18	166	274	166	100	87.37	60.58	89.42
19	162	303	150	92.59	82.43	49.51	84.03
20	153	225	115	75.16	88.91	51.11	87.07
Total	2221	3258	1863				
Average				82.98	89.35	61.48	88.52

*REM*_tot_ number of REM epochs in the test,* REM*_det_ number of REM epochs detected by the algorithm.* TP* true positives,* Sen* sensitivity,* Spe* specificity, *Sel * selectivity, *Acc* accuracy

The first stage of the algorithm uses *SEFd* to detect most of the REM epochs while the second stage uses *AP* and *RP* to eliminate false detections in the first stage. In order to illustrate this, the performance of the algorithm was quantified in both stages: it was run first using the *SEFd* feature only and then the *AP* and *RP* features were added to it. Results in Table 6 shows an increase in specificity, selectivity and accuracy when the *AP* and *RP* are used together with the *SEFd*. Furthermore, it can be seen that the number of false positives is reduced from 2534 to 1395 with the addition of these features. However this performance boost comes at the cost of a slight reduction in average sensitivity from 88.7 to 83% when the *AP* and *RP* features are added. Depending on the application, a suitable trade-off must be achieved to reduce the number of false positives up to a point where reduction in the number of true positives is acceptable. Conversely, both specificity and selectivity can be traded off to achieve higher sensitivity if higher number of false positives can be tolerated.

**Table 6:** Algorithm performance analysis at output of first and second stages

Features	TP	FP	TN	FN	Sen (%)	Spe (%)	Sel (%)	Acc (%)
*SEFd* only	1996	2534	10,437	225	88.67	80.52	48.40	81.91
*SEFd*, *AP* and *RP*	1863	1395	11,576	358	82.98	89.35	61.48	88.52

*TP* true positives,* FN* false negatives,* TN* true negatives,* FP* false positives, (numbers are total for 15 test subjects).* Sen* sensitivity,* Spe* specificity,* Sel* selectivity,* Acc* accuracy, (numbers are average for 15 test subjects)

A breakdown of the false detections in Table 7 shows in which sleep stages these false positives occur, as well as the fraction of each stage falsely scored as REM. Across the 15 test subjects, only 18.5% of the total Wake epochs are misclassified as REM. Amongst these, almost a quarter of false positives in Wake stage come from subject 8 alone. Similarly, about a third of total N1 epochs are misclassified as REM (424 out of a total of 1157 N1 epochs). Since N1 and REM have similarities in EEG, this is to be expected. It is however still a positive result since it does show a discriminatory ability that can be used to distinguish between REM and N1 stages using EEG. Only 431 out of the total 5936 N2 epochs are misclassified as REM (about 7%) where subject 7 contributes almost a fifth to the false positives in N2. Finally, only 7 N3 epochs across all 15 test subjects are misclassified by the algorithm as REM and 5 of those come from subject 7.

**Table 7:** Breakdown of all false detections in test database

Subject	TP	FN	TN	FP	FP_*W *_(W)	FP$_{N1}$ (N1)	FP$_{N2}$ (N2)	FP$_{N3}$ (N3)
6	186	1	698	111	48 (179)	25 (51)	38 (355)	0 (224)
7	107	24	703	178	50 (394)	31 (49)	92 (283)	5 (155)
8	120	42	643	164	119 (181)	41 (95)	4 (324)	0 (207)
9	124	7	926	60	5 (216)	33 (71)	22 (515)	0 (184)
10	113	33	845	40	23 (71)	8 (66)	8 (411)	1 (337)
11	203	9	731	65	1 (122)	6 (67)	57 (401)	1 (206)
12	52	35	862	12	6 (393)	3 (90)	3 (234)	0 (157)
13	88	1	839	179	69 (181)	73 (112)	37 (432)	0 (293)
14	105	58	828	13	0 (208)	5 (46)	8 (417)	0 (170)
15	92	31	693	21	1 (114)	20 (98)	0 (294)	0 (208)
16	105	42	761	59	4 (258)	23 (88)	32 (370)	0 (104)
17	137	25	700	122	60 (67)	20 (45)	42 (564)	0 (146)
18	166	0	747	108	54 (169)	40 (87)	14 (420)	0 (179)
19	150	12	718	153	17 (129)	70 (131)	66 (460)	0 (151)
20	115	38	882	110	76 (198)	26 (61)	8 (456)	0 (277)
Total	1863	358	11576	1395	533 (2880)	424 (1157)	431 (5936)	7 (2998)

FP$_{X}$(X) shows false positives in stage X and the total number of epochs from stage X in parentheses

*TP* true positives,* FN* false negatives,* TN* true negatives,* FP* false positives

The agreement rate between the algorithm and the visual scorer was also evaluated using Cohen’s kappa ($\kappa$) values. For the test data including all sleep stages $\kappa$ was found to be 0.61, representing substantial agreement according to Landis and Koch’s classification.^24^

### Fivefold Cross-Validation

The performance of the algorithm was also validated using fivefold cross-validation. The entire database was divided into five groups, each consisting of four subjects. On each iteration, the algorithm was trained using four groups (16 subjects) and tested using the remaining group (4 subjects). This results in an average sensitivity of 85.8% while specificity, selectivity and accuracy values are 89.3, 61.9 and 88.8% respectively. In comparison to the results obtained in “Test Results” section, the sensitivity value achieved is slightly higher while all other performance metrics are very similar. The detection results for individual subjects can be seen in Supplementary Material Table S1.

### Performance Comparison

There are very few single-channel EEG-based sleep scoring methods in literature. Our search revealed only three such methods^2^,^27^,^35^ detailing REM detection performance. It is difficult to compare the results of different algorithms due to the varying databases used to test each of them. However, since these methods also report their performance on the publicly available PhysioNet Sleep-EDF database,^33^ we evaluated our algorithm using the same database for a fair comparison.

This database consists of PSG recordings from 8 healthy subjects with two channels of EEG recorded for each. We used the Fpz-Cz channel only and evaluated the algorithm using leave-one-out cross validation (LOOCV) on over 8800 scored epochs across all 8 subjects (about 9 hours recording for each subject) with the exception of movement (MT) and unscored epochs.

The performance of our algorithm and those of other one-channel EEG-based methods on the same database for REM detection is shown in Table 8. The algorithm achieved similar sensitivity and selectivity, compared to others, while using only three features. If there are processing and power constraints attached with the system then the algorithm presented in this paper could be used to achieve REM detection performance that is similar to other methods using a much smaller number of features and a simple classifier. However, if there are no such limitations either of the methods listed in Table 8 would achieve similar results. Further, we used the Fpz-Cz channel to evaluate the algorithm’s performance since the main feature used in this work (*SEFd*) exhibits strongest discriminatory ability in the frontal channels. The other algorithms listed in Table 8 used the channel Pz-Oz because the this was closest to their algorithm requirements and gave the best results.

**Table 8:** Performance comparison with other single-channel EEG methods on PhysioNet Sleep-EDF database

	Method	No. of features	Classifier	Sen (%)	Sel (%)
This work	Spectral power	3	Thresholding	80.6	74.8
Ref. 27	MSE, AR model	21	LDA and contextual smoothing	85.4	78.8
Ref. 35	Spectral power	30	Neural network	82.3	–
Ref. 2	Spectral and temporal features	Multiple	Fuzzy classifier and contextual smoothing	63.0	91.7

## Discussion

Automatic detection of REM stages in sleep is desirable to aid in the development of a fully automated sleep staging system. The bulk of sleep staging is performed using EEG signals while EOG and EMG signals are generally required to mark REM stages. During the REM phases there are characteristic bursts of eye movements observed on EOG traces that are used to score them. However these eye movements are present for only up to 27% of the total REM sleep time.^25^ This suggests that EOG signals, albeit helpful, may not be able to detect all REM stage epochs. For a wearable sleep staging system, size and power are the main constraints. A reduction in the number of channels directly helps in power saving by reducing the amount of signals to process thereby minimising processor load and size and consequently improving battery life. It also leads to a physical system that is lighter in weight and easy to use. Identification of REM stage from one channel of EEG with reliable performance, therefore, could go a long way in system processing, power and size reduction.

In this paper, the difference between spectral edge frequencies (*SEF95* and *SEF50*) in the 8–16 Hz frequency band is introduced as a novel feature that exhibits clear discriminatory abilities for scoring REM epochs. On a test database of 15 subjects, this feature alone was able to detect 88.7% of the total REM epochs. The database was used as is, without removing any movement artefacts or stages, to reflect real world recording conditions. Absolute and relative powers in the same spectral band were used as added features to further analyze the candidate REM epochs at the first stage. This helped in reducing the number of false detections by more than 40%. The final two-stage algorithm resulted in sensitivity of 83% within a 95% confidence interval range of 81.4 to 84.5% for a total of 2221 test REM epochs while the Cohen’s kappa value showed substantial agreement between visual and automatic detection of REM. The algorithm also resulted in similar performance compared to other single-channel EEG-based methods when evaluated on the same database.

The algorithm achieved its highest detection performance using data from the frontal (Fp1-A2) channel. The performance degraded when the C3-A1 channel was used while it was worse using the O1-A2 channel. This suggests the the performance steadily reduces when moving away from the frontal region of the brain. This can be explained by the conclusions of Corsi-Cabrera* et al.* 8 on the fact that REM sleep exhibits uncoupled EEG activity between frontal and posterior regions of brain. Thus, features present in the frontal region during REM sleep may be completely absent in the posterior region. The close proximity of Fp1-A2 channel to the EOG could also result in some eye movement activity being picked up in the frontal EEG thus resulting in better performance.

The REM detection algorithm uses fixed thresholds to classify REM epochs for all test subjects. This simplifies the classification stage thus reducing the algorithm’s complexity. The use of patient-specific thresholds was also investigated. This resulted in the average sensitivity increasing to 90%, specificity 94%, selectivity 73% and accuracy of about 94%. The increase in sensitivity is a consequence of using patient-specific *SEFd* threshold that resulted in 132 more REM epochs being correctly identified. Adjusting the *AP* threshold reduced the number of false detections by almost 50% (down from 1395 to 752 epochs) thereby improving the overall selectivity. The most notable reduction is in the number of misclassified epochs in Wake stage followed by N1 and N2 stages. The mean and median averages and the standard deviation of all patient-specific thresholds are shown in Table 9. The mean and median values of the *SEFd* threshold are 4.54 and 4.5 Hz respectively which is close to the fixed threshold being used. For *AP*_max_ and *RP*_max_, both mean and median values are close to each other, but slightly less than the fixed threshold value used. For *RP*_min_ the difference between mean and median averages is the largest and both these values are lower than their fixed-threshold counterpart. The relative standard deviation is lowest for *SEF*th at 4% while for the other three thresholds it is between 12–17%. This suggests that the use of adaptive thresholds that can adjust to individual subjects can further improve the results and will be explored in future work. However, this improvement in performance will come at the cost of additional algorithm complexity. Nevertheless, the use of fixed thresholds still achieves a performance comparable to other algorithms thus highlighting the strength of the approach.

**Table 9:** Mean, median and standard deviation of the patient-specific thresholds

Threshold	Mean	Median	SD
SEFth (Hz)	4.54	4.50	0.18
AP_max_ (dB)	15.07	15.30	2.12
RP_max_ (dB)	−6.19	−6.60	0.75
RP_min_ (dB)	−13.41	−14.30	2.31

The REM detection algorithm presented here has several advantages. First, its performance is comparable to most of the methods in literature including those that use multiple EEG, EOG and EMG channels. Second, it uses a simple thresholding method with fixed thresholds to mark REM epochs in contrast to some other systems that use complex neural networks with a large input feature set. This low-complexity classifier is advantageous for portable and wearable systems with limited processing cycles and power budget. Third, results from automatic sleep staging systems of other research groups^12^,^14^,^28^ suggest overlap of REM stage with N1 in various feature spaces. These two stages have similar EEG and are difficult to differentiate as discussed in “Introduction” section. The feature used here also successfully distinguishes between the majority of N1 and REM epochs. About 63% of the total N1 epochs were correctly distinguished from REM despite their strong EEG similarities. The misclassification proportion in Wake stage was much smaller, at 18.5%. This is, even with the inclusion of the movement epochs (which are marked as Wake according to AASM rules). This number could go down further with the use of an artefact rejection method at the front end of the algorithm as well as using adaptive thresholding at the classification stage. About 7% of N2 epochs were wrongly detected as REM while only 7 out of 2998 N3 epochs were misclassified. The total number of false positive epochs was 1395 which may seem like a large number. However, the total epochs under test were 15192 from all stages of sleep. Considering this, the fraction of false positives is actually less than 10%. Ideally, the number of false positives should be even smaller. The use of patient-specific thresholds reduces it to 752 epochs (less than 5% false positives). Finally, the REM detection algorithm in this paper uses data from only one EEG channel and therefore keeps the data rate and processing load small.

Overall our investigations in this study illustrate that spectral edge frequency in the 8–16 Hz band of EEG can be a useful feature for the detection REM sleep phase. We have demonstrated this with a simple algorithm and achieved high accuracy from just one EEG channel. Although this algorithm showed a good performance, the main objective of this paper was not to present the best performing REM detection algorithm but to introduce and evaluate a novel feature that could be used with a simple algorithm or as an added feature in a different algorithm. The heuristic classifier used in this work is very simple and may not represent the most optimal approach. Other classifiers such as decision trees, support vector machines,* etc*. may result in an improved detection performance. Nevertheless, we believe that the results presented in this paper will be highly useful for EEG system designers by helping to reduce the number of channels, computational cost, device size and power consumption for future truly wearable and automated sleep staging systems.

## Electronic Supplementary Material

Below is the link to the electronic supplementary material.

Supplementary material 1 (PDF 22 kb)

## References

[1] Agarwal R, Gotman J (2001). Long-term EEG compression for intensive-care settings. IEEE Eng. Med. Biol. Mag..

[2] Berthomier C, Drouot X, Herman-Stoïca M, Berthomier P, Prado J, Bokar-Thire D, Benoit O, Mattout J, D’Ortho MP (2007). Automatic analysis of single-channel sleep eeg: validation in healthy individuals. Sleep.

[3] Bódizs R, Sverteczki M, Mészáros E (2008). Wakefulness-sleep transition: emerging electroencephalographic similarities with the rapid eye movement phase. Brain Res. Bull..

[4] Bruyneel, M., and V. Ninane. Unattended home-based polysomnography for sleep disordered breathing: current concepts and perspectives. *Sleep Med. Rev.* 18(4):341–347, 2014. 10.1016/j.smrv.2013.12.002 24388970

[5] Charbonnier S, Zoubek L, Lesecq S, Chapotot F (2011). Self-evaluated automatic classifier as a decision-support tool for sleep/wake staging. Comput. Biol. Med..

[6] Chesson AL, Berry RB, Pack A (2003). Practice parameters for the use of portable monitoring devices in the investigation of suspected obstructive sleep apnea in adults. Sleep.

[7] Coffin M, Sukhatme S (1997). Receiver operating characteristic studies and measurement errors. Biometrics.

[8] Corsi-Cabrera M, Miro E, del Rio-Portilla Y, Perez-Garcia E, Villanueva Y, Guevara MA (2003). Rapid eye movement sleep dreaming is characterized by uncoupled eeg activity between frontal and perceptual cortical regions. Brain Cognition.

[9] Corsi-Cabrera M, Munoz-Torres Z, del Rio-Portilla Y, Guevara MA (2006). Power and coherent oscillations distinguish rem sleep, stage 1 and wakefulness. Int. J. Psychophysiol..

[10] Danker-Hopfe H, Anderer P, Zeitlhofer J, Boeck M, Dorn H, Gruber G, Heller E, Loretz E, Moser D, Parapatics S, Saletu B, Schmidt A, Dorffner G (2009). Interrater reliability for sleep scoring according to the rechtschaffen & kales and the new aasm standard. J. Sleep Res..

[11] Ebrahimi, F., M. Mikaeili, E. Estrada, and H. Nazeran. Automatic sleep stage classification based on eeg signals by using neural networks and wavelet packet coefficients. In: IEEE EMBC. Canada: Vancouver, 2008. 10.1109/IEMBS.2008.4649365 19162868

[12] Ebrahimi, F., M. Mikaili, E. Estrada, and H. Nazeran. Assessment of itakura distance as a valuable feature for computer-aided classification of sleep stages. In: IEEE EMBC. Lyon, 2007. 10.1109/IEMBS.2007.4353035 18002701

[13] Estrada, E., H. Nazeran, J. Barragan, J. R. Burk, E. A. Lucas, and K. Behbehani. Eog and emg: two important switches in automatic sleep stage classification. In: IEEE EMBC. New York, 2006. 10.1109/IEMBS.2006.260075 17946514

[14] Estrada, E., H. Nazeran, F. Ebrahimi, and M. Mikaeili. Eeg signal features for computer-aided sleep stage detection. In: IEEE EMBS NER. Antalya, 2009.

[15] Flemons WW, Douglas NJ, Kuna ST, Rodenstein DO, Wheatley J (2004). Access to diagnosis and treatment of patients with suspected sleep apnea. Am. J. Respir. Crit. Care Med..

[16] Günes, S., K. Polat, and C. Yosunkaya. Efficient sleep stage recognition system based on eeg signal using k-means clustering based feature weighting.* Expert Syst. Appl.* 37(12):7922–7928, 2010.

[17] Hanaoka, M., M. Kobayashi, and H. Yamazaki. Automated sleep stage scoring by decision tree learning. In: IEEE EMBC. Chicago, 2000.

[18] Held CM, Heiss JE, Estévez PA, Perez CA, Garrido M, Algarín C, Peirano P (2006). Extracting fuzzy rules from polysomnographic recordings for infant sleep classification. IEEE Trans. Biomed. Eng..

[19] Horne J (2013). Why rem sleep? clues beyond the laboratory in a more challenging world. Biol. Psychol..

[20] Iber C, Ancoli-Israel S, Chesson A, Quan S (2007). The AASM manual for the scoring of sleep and associated events: rules, terminology and technical specifications.

[21] Indursky P, Rotenberg V (1998). Change of mood during sleep and rem sleep variables. Int. J. Psychiatry Clin. Pract..

[22] Iranzo A, Molinuevo JL, Santamaría J, Serradell M, Martí MJ, Valldeoriola F, Tolosa E (2006). Rapid-eye-movement sleep behaviour disorder as an early marker for a neurodegenerative disorder: a descriptive study. Lancet Neurol..

[23] Kempfner, J., P. Jennum, M. Nikolic, J. A. E. Christensen, and H. B. D. Sorensen. Automatic rem sleep detection associated with idiopathic rem sleep behavior disorder. In: IEEE EMBC. San Diego, 2012. 10.1109/IEMBS.2011.6091498 22255722

[24] Landis J, Koch G (1977). The measurement of observer agreement for categorical data. Biometrics.

[25] Leclair-Visonneau L, Oudiette D, Gaymard B, Leu-Semenescu S, Arnulf I (2010). Do the eyes scan dream images during rapid eye movement sleep? evidence from the rapid eye movement sleep behaviour disorder model. Brain.

[26] Liang SF, Kuo CE, Hu YH, Cheng YS (2012). A rule-based automatic sleep staging method. J. Neurosci. Methods.

[27] Liang SF, Kuo CE, Hu YH, Pan YH, Wang YH (2012). Automatic stage scoring of single-channel sleep eeg by using multiscale entropy and autoregressive models. IEEE Trans. Instrum. Meas..

[28] Ma, Q., X. Ning, J. Wang, and J. Li. Sleep-stage characterization by nonlinear eeg analysis using wavelet-based multifractal formalism. In: IEEE EMBC. Shanghai, 2005. 10.1109/IEMBS.2005.1615475 17281245

[29] Mellman TA, Bustamante V, Pigeon WR, Nolan B (2002). Rem sleep and the early development of posttraumatic stress disorder. Am. J. Pschiatry.

[30] Nykamp K, Rosenthal L, Folkerts M, Roehrs T, Guido P, Roth T (1998). The effects of rem sleep deprivation on the level of sleepiness/alertness. Sleep.

[31] Park, H., K. Pa, and D. U. Jmn. Hybrid neural-network and rule-based expert system for automatic sleep stage scoring. In: IEEE EMBC. Chicago, 2000.

[32] Perkins NJ, Schisterman EF (2006). The inconsistency of optimal cut-points using two roc based criteria. Am. J. Epidemiol.

[33] PhysioNet: Sleep-edf database (2013). http://www.physionet.org/physiobank/database/sleep-edf/.

[34] Rechtschaffen, A., and A. Kales. (eds.). A manual of standardized terminology, techniques and scoring system for sleep stages of human subjects. Washington, DC: Public Health Service, U.S. Government Printing Office, 1968.

[35] Ronzhina M, Janousek O, Kolarova J, Novakova M, Honzik P, Provaznik I (2012). Sleep scoring using artificial neural networks. Sleep Med. Rev..

[36] Ruehland WR, O’Donoghue FJ, Pierce RJ, Thornton AT, Singh P, Copland JM, Stevens B, Rochford PD (2011). The 2007 aasm recommendations for eeg electrode placement in polysomnography: impact on sleep and cortical arousal scoring. Sleep.

[37] Shambroom JR, Fábregas SE, Johnstone J (2012). Validation of an automated wireless system to monitor sleep in healthy adults. J. Sleep Res..

[38] Sleep Sos Report: The Impact of Sleep on Society. The Sleep Alliance, 2007.

[39] Tian, J. Y., and J. Q. Liu. Automated sleep staging by a hybrid system comprising neural network and fuzzy rule-based reasoning. In: IEEE EMBC. Shanghai, 2005. 10.1109/IEMBS.2005.1615368 17281138

[40] Uchida S, Maloney T, Feinberg I (1994). Sigma (12–16 hz) and beta (20–28 hz) eeg discriminate nrem and rem sleep. Brain Res..

[41] University of MONS—TCTS Laboratory: The DREAMS Subjects Database (2013). http://www.tcts.fpms.ac.be/devuyst/Databases/DatabaseSubjects/.

[42] Virkkala, J., R. Velin, S. Himanen, A. Varri, K. Muller, and J. Hasan. Automatic sleep stage classification using two facial electrodes. In: IEEE EMBC. Vancouver, 2008. 10.1109/IEMBS.2008.4649489 19162992

[43] Vogel GW, Vogel F, McAbee RS, Thurmond AJ (1980). Improvement of depression by rem sleep deprivation. Arch. Gen. Pschiatry.

