# Supplementary material for: A Low Computational Cost Algorithm for REM Sleep Detection Using Single Channel EEG
Source: Ann Biomed Eng. 2014 Aug 12;42(11):2344–59. doi: 10.1007/s10439-014-1085-6 (PMC4204008; doi:10.1007/s10439-014-1085-6)
Supplement: Supplementary file 1 — Supplementary material 1 (PDF 22 kb) [file 10439_2014_1085_MOESM1_ESM.pdf]

A Low Computational Cost Algorithm for REM Sleep Detection using Single Channel EEG

Syed Anas Imtiaz and Esther Rodriguez-Villegas

Table S1 Performance of algorithm using five-fold cross-validation.

Subject	REM_{tot}	REM_{det}	TP	Sen (%)	Spe (%)	Sel(%)	Acc(%)
1	113	158	103	91.15	93.51	65.19	93.24
2	122	242	119	97.54	85.75	49.17	87.21
3	212	224	189	89.15	95.6	84.38	94.25
4	155	324	146	94.19	80.18	45.06	82.24
5	196	279	195	99.49	90.08	69.89	91.85
6	187	297	186	99.47	86.28	62.63	88.76
7	131	285	107	81.68	79.8	37.54	80.04
8	162	284	120	74.07	79.68	42.25	78.74
9	131	182	123	93.89	94.02	67.58	94
10	146	150	111	76.03	95.59	74	92.82
11	212	267	203	95.75	91.96	76.03	92.76
12	87	61	50	57.47	98.74	81.97	95.01
13	89	283	88	98.88	80.84	31.1	82.29
14	163	130	117	71.78	98.45	90	94.12
15	123	114	93	75.61	97.06	81.58	93.91
16	147	150	99	67.35	93.78	66	89.76
17	162	259	137	84.57	85.16	52.9	85.06
18	166	274	166	100	87.37	60.58	89.42
19	162	303	150	92.59	82.43	49.51	84.03
20	153	225	115	75.16	88.91	51.11	87.07
avg				85.79	89.26	61.92	88.83
Total	3019	4491	2617				
Average				85.79	89.26	61.92	88.83

REM_{tot} - number of REM epochs in the test; REM_{det} - number of REM epochs detected by the algorithm.

TP - true positives; Sen - sensitivity; Spe - specificity; Sel - selectivity; Acc - accuracy.

Department of Electrical and Electronic Engineering
Imperial College London, UK
anas.imtiaz@imperial.ac.uk
